# Human–pet interaction and well-being in mid-to-later adulthood: moderating roles of neuroticism and anxious attachment

**DOI:** 10.1186/s12889-025-24571-6

**Published:** 2025-10-16

**Authors:** Ya-Ling Wang, Ching-Han Tsai, Chih-Chi Liu

**Affiliations:** 1 Department of Adult and Continuing Education, National Taiwan Normal University, 129 Section 1 Heping East Road, Taipei City 106, Taipei, Taiwan; 2 Department of Long-Term Care, Hsin Sheng Junior College of Medical Care and Management, No. 418, Sec. Gaoping, Zhongfeng Rd., Longtan Dist., Taoyuan City 325, Taoyuan, Taiwan

**Keywords:** Anxious attachment, Neuroticism, Pet attachment, Pet interaction, Well-being

## Abstract

**Background:**

Nowadays, many middle-aged and older adults are starting to keep pets. However, previous studies have yielded divergent results regarding the well-being of middle-aged and older adults who keep pets. Therefore, this study aimed to (1) explore how individual differences in well-being are related to the interaction between middle-aged and older adults and their pets, (2) examine how neuroticism may moderate the relationship between the time middle-aged and older adults spend with their pets and their well-being, (3) investigate how anxious attachment orientation may moderate the relationship between the time middle-aged and older adults spend with their pets and their well-being. The subjects of the research were middle-aged and older adults over 45 years old who raised dogs or cats in Taiwan.

**Methods:**

The study sampled 292 subjects (232 valid), of whom 84% were women. This study used regression analysis to explore the correlation and interaction between variables.

**Results:**

Neuroticism significantly moderated the relationship between pet interaction time and well-being among middle-aged and older adults. Higher neuroticism was associated with decreased well-being as interaction time increased, while lower neuroticism showed no such effect. A marginally significant moderation effect also suggested that anxious attachment orientation may influence this relationship.

**Conclusions:**

The findings highlight the role of psychological traits in shaping the well-being effects of pet interaction. While increased interaction supports well-being in those with low anxious attachment, it may be detrimental for individuals with high anxious attachment. These results underscore the need to consider psychological differences when evaluating the benefits of pet companionship.

## Introduction

According to Taiwan’s biennial cat and dog population survey, the number of pet dogs and cats increased by 10% from 2017 to 2019, reaching approximately 2.3 million. This growth reflects a rising trend in pet guardianship among households [1, 2]. In Taiwan, the number of cats and dogs may exceed that of children aged 15 and below, reflecting the increasingly prominent presence of companion animals in households. Among middle-aged and older adults, many view pets not only as part of the family, but also as significant sources of emotional connection and everyday companionship [4]. With the aging population, research has begun to focus on the benefits of keeping pets for middle-aged and older adults. For example, a study by Friedmann et al. [3] surveyed middle-aged and older adults over the age of 50 and found that 82% of the participants had kept pets. Additionally, the participants indicated that their reasons for keeping pets included enjoyment (80%) and companionship (66%). This trend reflects the growing importance of pets in providing companionship and emotional support, especially as people enter later stages of life.

The human-animal bond is reinforced by the belief that pets can sense and respond to their caregivers’ emotions, providing comfort during difficult times. Meier & Maurer [5] found that pets provide important companionship for older adults, especially those living alone. However, their study also revealed that certain subgroups—such as individuals with lower educational attainment or those struggling to make ends meet—may face challenges related to pet care, including financial strain or caregiving demands. Dogs, for instance, may exhibit problematic behaviors, including furniture damage, disobedience, or aggression, which can be stressors for caregivers [6]. Applebaum et al. [7] further highlighted that while pets provided emotional support during the COVID-19 pandemic, they also introduced challenges such as financial strain and difficulties in securing pet care supplies. While pets enhance companionship and emotional well-being, the responsibilities of caregiving may not always lead to improved quality of life.

Pet guardianship among middle-aged and older adults involves a dynamic interplay of benefits and challenges [8–10, 98, 99]. On the one hand, pets can offer companionship, emotional support, and promote physical activity, all of which are associated with enhanced well-being. On the other hand, challenges such as financial burden, caregiving responsibilities, and emotional strain—particularly in the case of pet illness or loss—can complicate this relationship. Research has highlighted how the effects of pet guardianship may vary depending on factors such as the guardian’s attachment to their pet and individual personality traits. For instance, Wells and Treacy [8] emphasize the complexity of the links between pet attachment, personality, and well-being, while Li et al. [9] note that financial strain can offset the potential benefits of pet companionship. Building on these findings, the present study aims to explore the underlying factors contributing to these mixed outcomes and to offer guidance for middle-aged and older adults who are considering pet adoption, helping them enjoy the emotional rewards of companionship while minimizing potential stressors.

While prior research has acknowledged both the benefits and challenges of pet guardianship, it remains unclear why some middle-aged and older adults experience enhanced well-being through pet companionship while others do not. This suggests that additional psychological or relational mechanisms may moderate this relationship, highlighting the need to further examine the underlying factors that shape the impact of daily pet interaction on psychological well-being. Therefore, this study aims to explore the mechanisms through which pet companionship may influence well-being among middle-aged and older adults. The findings are expected to provide practical suggestions for those considering pet adoption, helping them enhance their well-being through positive human–animal interactions. Based on the research objectives, the questions this study seeks to answer are: (1) Can neuroticism traits moderate the relationship between middle-aged and older adults’ interaction time with their pets and their well-being? (2) Can anxious attachment moderate the relationship between middle-aged and older adults’ interaction time with their pets and their well-being?

### Pet interaction among middle-aged and older adults

Middle-aged and older adulthood is often marked by a series of life transitions, such as changes in family roles, retirement, or shifts in social networks [13, 111]. While these transitions may bring new opportunities for personal growth and autonomy, they may also involve adjustments in emotional and social life. In such situations, pets may offer a relatively stable source of companionship and emotional support. Previous studies have noted that pet companionship may help enhance life satisfaction [11], provide emotional regulation [12], and offer a sense of routine during times of change. It has become common for middle-aged and older adults to keep pets [14]. Therefore, this study focuses on exploring how interaction with pets is associated with well-being among individuals aged 45 and above.

Following Thomas’s [15] definition, pets are animals kept in homes, given names by their parents, and not intended for consumption. According to recent studies, Lass-Hennemann et al.‘s study [16] highlight the role of pets in forming emotional attachment relationships with their human caregivers. While emotional support and emotional attachment are distinct constructs—since one can receive emotional support without forming a deep attachment—pets provide a unique form of emotional support by offering consistent companionship, non-judgmental affection, and responsiveness to their caregivers’ emotional states. This distinction is crucial in understanding how pet companionship influences well-being beyond traditional social support systems. Additionally, research from Borgi and Cirulli [17] discussed pets in the context of providing social and emotional support, particularly during times of adversity such as the COVID-19 pandemic. In this study, the term pets refers to domesticated animals—primarily dogs and cats—that are kept for companionship and caregiving purposes rather than for utilitarian or commercial use. Beyond their physical presence, pets are often seen as emotionally significant others in the daily lives of their guardians, contributing to feelings of comfort, security, and relational attachment. This definition serves as a foundation for exploring how human–animal bonds may influence psychological well-being in later life. While pets can contribute to their caregivers’ well-being, this is not a defining criterion; rather, their presence and interactions with their caregivers form the basis of this study. People often treat their pets as they would children or infants [18]. Thus, in this study, pets are defined as dogs and cats, as these are the most commonly kept types of pets in households. Dogs and cats can provide companionship to middle-aged and older adults, require attention and care, can form emotional bonds with humans, and engage in interactive behaviors.

Later life is often accompanied by changes in social relationships and physical functioning. These changes may include a shrinking of social networks, decreased mobility, or greater vulnerability to health-related issues [19, 100]. Research suggests that pet companionship can promote physical activity, support mobility, and encourage outdoor engagement, which may benefit cardiovascular and respiratory health [20–23]. Beyond physical health, pets provide emotional support and engagement, helping to mitigate loneliness and sensory decline. Watching animals offers visual stimulation, while tactile interactions may help preserve sensory function. Pets can also enhance safety awareness, providing timely alerts in emergencies such as fire or gas leaks [24]. Additionally, caring for pets promotes regular activity, which may contribute to lower blood pressure and overall well-being in older adults [23]. These benefits highlight the multifaceted role of pet companionship in supporting both physical and emotional health in later life.

Research indicates that keeping pets requires time for companionship. When it comes to keeping dogs, spending time with them, particularly through walking [25], increases the outdoor activity time for middle-aged and older adults, subsequently enhancing their health. Moreover, if partners or other family members cannot provide sufficient companionship, the extended periods of interaction between middle-aged and older pet parents with their pets can also be a way to improve their physical and mental health [26, 27]. However, pet parents need to allocate time for activities like walking, feeding, and bathing their pets, which might sacrifice the time they could spend with family or on achieving personal life goals [28, 101]. From the above, some studies suggest that companionship and caring for pets bring well-being and promote health, while others indicate that it can be stressful.

In summary, as individuals age, there is often a natural decline in physical health. Living with pets may help promote physical activity among middle-aged and older adults, which could support healthier aging. However, the impact of devoting time and caring for pets can vary depending on factors such as the duration of companionship, time spent on pet care they have. Extended periods of companionship with pets can increase physical activity and promote the physical health of middle-aged and older adults [29]. However, this prolonged companionship may also compress their personal schedules and cause stress. As noted above, prior studies have reported inconsistent findings regarding the effects of the duration of pet companionship on well-being. This study aims to further examine these dynamics and clarify the potential relationship.

### Psychological well-being of middle-aged and older adults

This study employs Ryff’s [30] psychological well-being as the dependent variable. Psychological well-being represents an individual’s positive functioning in areas such as life satisfaction, connections with others, and life goals [30]. Middle-aged and older adults with higher psychological well-being have a lower probability of hospitalization [31] and reduced occurrence of depression and anxiety [32, 33]. Ryff [34] proposed a lifespan theory that considers an individual’s psychological well-being at different stages of life, with successful aging as its core concept. Recent research using the Psychological Well-being Scale has provided valuable insights into well-being in middle-aged and older adults. This study adopts this framework, as it aligns with the psychological characteristics of individuals in this life stage.

Enhancing the well-being of middle-aged and older adults is a crucial topic today [39, 102]. Delhom et al. [35] found that life satisfaction, defined as a sense of living and values, which represents the realization of psychological well-being, effectively predicts psychological well-being in middle-aged and older adults. Smith and Hollinger-Smith [36] showed that middle-aged and older adults who have the ability to seek positive experiences and view life with gratitude experience significant improvements in life satisfaction and psychological well-being. Emotional intelligence (EI) is closely related to well-being [37]. Among the six dimensions of psychological well-being, emotional intelligence has a smaller predictive power for environmental mastery [35]. However, it allows middle-aged and older adults to effectively and strategically cope with environmental changes, leading to rapid emotional recovery and improved mental health. Resilience positively predicts psychological well-being and positive emotions significantly promote the well-being of middle-aged and older adults [36]. Middle-aged and older adults’ attitudes towards aging are also related to well-being. Lau and Cheung [38] found that middle-aged and older adults who mention more grateful events rather than negative ones in their diaries experience reduced anxiety about death, improved emotional problems, and enhanced well-being. Guided by a multidimensional view of well-being, the present study focuses on psychological well-being as the primary outcome of interest.

### Relationship between keeping pets among middle-aged and older adults and well-being

Existing studies suggest that the effects of pet companionship on well-being are not universally positive, but rather vary depending on individual and contextual factors. Pets provide emotional and psychological support, reducing loneliness and isolation [40]. They offer companionship, a sense of purpose, and cognitive benefits, such as alleviating dementia symptoms [41–43]. These factors contribute to higher life satisfaction and improved well-being [42].

However, some research suggests that pet companionship does not necessarily improve mental health [44]. Lonely individuals may seek companionship through pets, but this does not always yield the desired benefits [45, 46]. Additionally, strong emotional dependence on pets can lead to intensified grief upon their loss [47]. Behavioral challenges, including aggression, disobedience, and excessive barking, can also negatively impact well-being [48]. Moreover, pets pose physical risks, such as increasing the likelihood of falls and fractures among older adults [49]. Previous research suggests that the effects of pet companionship on well-being are not uniform. While some individuals benefit from companionship and increased activity, others may experience emotional or physical burdens. These mixed outcomes point to the potential role of psychological factors, which are examined in the following section.

### Neuroticism, attachment and pet interaction in middle-aged and older adults

Retirement often leads to a loss of purpose, contributing to feelings of emptiness and loneliness [50]. As a response, many middle-aged and older adults turn to pets for emotional support [51]. While pet companionship is generally seen as beneficial, studies suggest it can also introduce psychological stress, potentially reducing overall well-being [44, 47, 48, 52]. The impact of pet companionship depends on the psychological traits of individuals, particularly neuroticism and anxious attachment, which may contribute to emotional instability and interpersonal difficulties.

Individuals with high attachment anxiety often exhibit neurotic traits, leading to persistent worry, self-doubt, and heightened sensitivity in relationships [53, 54]. They frequently seek reassurance, struggle with emotional regulation, and experience conflict in interpersonal relationships [55, 56]. These tendencies may extend to their pets, affecting both their caregiving experience and overall well-being.

Personality traits also influence pet behavior and satisfaction. For example, pet parents with high neuroticism are more likely to have pets displaying aggression and behavioral problems [57]. Similarly, individuals with anxious attachment tend to experience lower satisfaction in pet relationships, especially when their pets exhibit undesired behaviors [58]. Moreover, anxious attachment can lead to emotional dependence, resulting in anger, self-blame, and tension when interacting with pets [59].

In summary, individuals with high neuroticism and anxious attachment may transfer negative emotions to their pets, turning pet companionship into a source of stress rather than well-being. This study posits that neuroticism and anxious attachment moderate the relationship between pet interaction time and well-being in middle-aged and older adults.

### Moderation effects of neuroticism traits on the relationship between keeping pets and well-being in middle-aged and older adults

Neuroticism is the most predictive variable for psychological well-being, life satisfaction, and interpersonal relationships [60–64]. Neuroticism is one of the Big Five personality traits, also known as emotional instability. Individuals with high scores in neuroticism experience more variability in negative emotion in everyday life [103]. They often experience emotions such as anger, worry, depression, fear, frustration, anxiety, jealousy, guilt, loneliness, and guilt. Neuroticism is negatively correlated with emotional intelligence (EQ), which includes emotional regulation, motivation, and interpersonal relationship skills [65]. Participation in interpersonal relationships and social activities is associated with increased life satisfaction and well-being. However, individuals with high neuroticism often struggle in handling interpersonal relationships, leading to lower levels of well-being. Studies by Sun et al. [64] and Meléndez et al. [66] have consistently shown negative associations between neuroticism and all dimensions of psychological well-being. Given their emotional instability and propensity for negative emotions, high neuroticism in middle-aged and older adults often results in poor performance in aspects of psychological well-being such as “positive relations with others,” “environmental mastery,” and “self-acceptance.”

Wells and Rodi [44] found that pet ownership did not necessarily compensate for social isolation in older adults, suggesting that the emotional impact of pets may not be uniformly positive and may depend on individual expectations and contexts. Individuals high in neuroticism are generally more prone to emotional reactivity, including anger and frustration, particularly when confronted with situations that deviate from their expectations [65]. Highly neurotic middle-aged and older adults are also more sensitive to the negative effects brought about by pets [64, 66].

Based on the literature discussed above, it can be inferred that individuals with high neuroticism, when keeping pets, tend to worry and fear more than they anticipate. When companion animals exhibit disobedient or problematic behavior, some pet guardians may perceive this as a source of emotional distress, particularly if they feel responsible for their pet’s actions. Research has shown that owners of pets with behavioral issues often report negative emotions such as frustration, sadness, guilt, and anxiety, as well as a sense of failure in their caregiving role. These emotional responses may be intensified when the outcomes of training or caregiving do not meet their expectations [104]. When pets don’t meet their expectations, they may feel anger and anxiety. Hence, this study proposes Hypothesis 1 (H1):

#### H1

Neuroticism moderates the relationship between interaction time with pets and well-being. The higher the neuroticism trait of middle-aged and older adults, the longer their interaction time with pets, the lower their well-being; conversely, the lower the neuroticism trait of middle-aged and older adults, the longer their interaction time with pets, the higher their well-being.

### Moderation effects of anxious attachment on the relationship between keeping pets and well-being in middle-aged and older adults

Past research has shown a significant relationship between attachment tendencies and well-being [61, 67–69]. When individuals form attachments to pets, although the attachment object requires care, there are similarities between this attachment and interpersonal attachment. For example, Levinson [9] found that pets provide humans with unconditional acceptance and emotional support, making individuals feel loved. Pets are loyal, non-judgmental, and non-comparative, which can lead to dependency on their parents, fostering attachment relationships. Attachment to pets can also be divided into two dimensions: anxiety and avoidance. When attachment anxiety is high, pet parents often doubt whether their pets love them and need repeated reassurance of their pets’ affection. They may also feel that their pets are not close enough to them. When they need to leave their pets alone at home, they worry about their pets’ loneliness and fear losing them due to the separation [59]. Those with high attachment anxiety have many worries and anxieties when raising pets, leading to reduced well-being. On the other hand, individuals with high attachment avoidance tend to maintain a distance from their pets. They may feel uncomfortable when their pets approach them [59]. Therefore, this study suggests that individuals with an avoidance attachment style are less likely to keep pets and are less likely to experience psychological changes due to spending time with their pets. Additionally, the relationship between avoidance attachment and neuroticism is weaker [56, 68]. As a result, this study only uses the anxiety attachment dimension as a moderating variable.

The duration of interaction between middle-aged and older pet parents and their pets can influence attachment relationships. Prolonged interaction with pets can lead to higher attachment, and if this attachment is characterized by anxiety, this may reduce the well-being of middle-aged and older adults. Hainlen et al. [70] found that anxious attachment individuals have fewer positive emotions, poorer forgiveness abilities towards others, and are more sensitive to social rejection and stress [71]. Furthermore, anxious attachment individuals take longer to recover from trauma [72], indicating more difficulties in interpersonal relationships. Due to prolonged exposure to negative emotions, highly anxious attachment individuals tend to take more time to ease their emotions during setbacks. Based on the aforementioned research, the study suggests that highly anxious attachment individuals are less tolerant of their pets’ mistakes when raising them. They may lack confidence and often doubt their pets’ loyalty. Therefore, the increased stress from mistrust in their relationship with their pets, along with the anxiety of losing their pets someday, can overshadow the well-being they currently derive from their pets. Hence, this study hypothesizes that highly anxious attachment among middle-aged and older adults, when keeping pets, is likely to reduce their well-being. Based on the above literature, this study proposes Hypothesis 2 (H2):

#### H2

Attachment anxiety moderates the relationship between interaction time with pets and well-being. The higher the attachment anxiety among middle-aged and older adults, the longer their interaction time with pets, the lower their well-being; conversely, the lower the attachment anxiety among middle-aged and older adults, the longer their interaction time with pets, the higher their well-being.

Building on previous research, which shows that attachment tendencies and neuroticism are linked to interpersonal relationships and overall well-being, this study explores whether these associations extend to relationships between humans and pets. While past studies have predominantly focused on human interactions, this research seeks to understand if similar patterns emerge in human-pet relationships and how these connections might influence well-being.

## Method

This study adopted a cross-sectional quantitative survey design to explore the relationships between pet ownership, pet interaction, and psychological well-being among middle-aged and older adults. Participants were recruited through online convenience sampling, primarily via Facebook. Facebook was selected as the main distribution platform because it is widely used among middle-aged and older adults in Taiwan, allowing for efficient access to the target population.

### Participants

This study distributed an electronic questionnaire (Google Forms) and employed purposive sampling. The research focused on middle-aged and older adults in Taiwan aged 45 and above who have cats or dogs as pets. The researchers posted recruitment messages in Facebook pet groups to reach middle-aged and older adults who have pets. In the pilot phase, the study recruited 85 eligible middle-aged and older adults, resulting in 74 valid responses, with 75.7% being female (18 males and 56 females). During the formal data collection phase, the study recruited a total of 292 middle-aged and older adults aged 45 and above. After data screening, 60 responses were excluded due to incomplete responses, resulting in a final sample of 232 valid responses, with 83.6% being female (38 males and 194 females). Among the 232 valid responses, 199 participants (85.8%) were 45–64 years old, and 33 participants (14.2%) were 65 years old and above. To ensure the suitability of the survey instrument for middle-aged and older adults, a pilot study was first conducted. The aim of the pilot phase was to assess the internal consistency and factor structure of the measurement tool. Based on the results of the pilot study, necessary adjustments were made to improve clarity and reliability. The formal data collection phase was then carried out using the revised version of the instrument. Although both phases followed the same procedures—including online recruitment via social media platforms, administration through Google Forms, and presentation of informed consent prior to participation—only the data from the formal phase were included in the statistical analyses reported in this study.

### Measure

The data collection consists of five main sections: Basic Information, Pet Interaction Scale, Well-being Scale, Neuroticism Scale, and Pet Attachment Scale. Except for the Basic Information section, all four scales were subjected to factor analysis in the pilot phase to confirm their construct validity. Due to the interrelatedness of dimensions within each scale, factor analysis was conducted using oblique rotation with the Promax rotation method. Principal axis factor analysis was employed for this purpose. The following sections provide detailed descriptions of each scale:

#### Basic information

This section includes demographic information (gender, age, retirement status, marital status, cohabitation status) and pet-related data (types and numbers of pets kept, pet ages, duration of keeping pets, whether you are the primary caregiver for the pets).

#### Pet interaction scale

The Pet Interaction Scale was developed by the researchers for this study. Its suitability for factor analysis was confirmed through Bartlett’s sphericity test (χ² = 562.913, df = 55, *p* <.001) and a KMO value of 0.72, indicating adequate sampling adequacy. Exploratory Factor Analysis (EFA) extracted three factors, with items below a 0.40 factor loading or misaligned with the original construct removed [76], such as those related to play frequency or pet accompaniment during errands.

The first factor (Interactive Care) included items reflecting one-sided caregiving behaviors, where middle-aged and older adults interacted with their pets primarily as caregivers (factor loadings: 0.41–0.72). The second factor (Interactive Companionship) captured mutual interactions between pet parents and their pets, where both actively engaged with each other (factor loadings: 0.67–0.99). The third factor (Non-Interactive Companionship) represented mutual awareness without direct interaction, where the pet and owner maintained a relationship through indirect means (factor loadings: 0.90, 0.91). Together, these factors explained 57.67% of the variance. The scale demonstrated acceptable reliability, with Cronbach’s alpha ranging from 0.59 to 0.81. The Cronbach’s alpha coefficient for the Interactive Care subscale was 0.59, indicating relatively lower internal consistency. Nevertheless, the subscale was retained for further analysis due to its theoretical relevance and its prior use in related research. Responses were measured on a 7-point Likert scale, where 1 indicated low interaction frequency and 7 indicated high interaction frequency.

#### Well-being scale

This study utilized the Psychological Well-being Scale developed by Ryff (1989a), which measures six dimensions: self-acceptance, positive relations, autonomy, environmental mastery, purpose in life, and personal growth. Given potential cultural differences, we adopted a Chinese version of the scale, referenced by Chiang et al. [73], and conducted an exploratory factor analysis (EFA) to assess its structure in our sample of middle-aged and older adults.

EFA results supported a three-factor structure, aligning with the original scale but reflecting conceptual refinements based on factor loadings. Factor 1: Combined personal growth and self-acceptance (4 items, factor loadings: 0.50–0.94), named “Self-Growth and Acceptance.” Factor 2: Represented positive relations with others (3 items, factor loadings: 0.51–0.92). Factor 3: Captured life purpose (5 items, factor loadings: 0.42–0.89).

These three factors explained 58.2% of the variance, and the scale demonstrated good reliability (Cronbach’s α = 0.78–0.84). Bartlett’s test confirmed suitability for factor analysis (χ2 = 1494.952, df = 66, *p* <.001), with a KMO value of 0.894 [74, 75]. The final 30-item scale used a 7-point Likert scale (1 = strongly disagree, 7 = strongly agree), with reverse scoring for negatively worded items and scores averaged per dimension.

#### Neuroticism scale 

This study utilized the Traditional Chinese version of the International English Big-Five Mini-Markers scale, developed by Teng et al. [77], based on Thompson’s [78] adaptation of Saucier’s [79] scale. Only neuroticism-related items were extracted for analysis.

Bartlett’s sphericity test confirmed suitability for factor analysis (χ² = 418.113, df = 55, *p* <.001), with a KMO measure of 0.812 [74, 75]. Exploratory Factor Analysis (EFA) initially suggested a two-factor structure, but items with factor loadings below 0.40 or misaligned with the original construct, such as “impatient,” “patient,” “unworried,” and “melancholic,” were removed. The final analysis retained four items (factor loadings: 0.47–0.89), supporting a single-factor solution that provided a more reliable and interpretable measurement.

The retained “Neuroticism” factor explained 55.3% of the variance, with a Cronbach’s alpha of 0.69, indicating acceptable reliability. The scale used a 7-point Likert scale (1 = strongly disagree, 7 = strongly agree), with reverse scoring applied where necessary, followed by summing and averaging the scores, resulting in a final neuroticism score ranging from 1 to 7.

#### Pet attachment scale 

This study employed the Pet Attachment Questionnaire, adapted by Zilcha-Mano et al. [45] based on adult attachment theory. Bartlett’s sphericity test (χ² = 623.024, df = 78, *p* <.001) and a KMO measure of 0.802 confirmed its suitability for factor analysis [60, 75].

Exploratory Factor Analysis (EFA) extracted two factors, with items below a 0.40 factor loading or misaligned with the original construct (e.g., “Sometimes I find my pet annoying”) removed. The first factor (7 items, factor loadings: 0.60–0.77) measured anxiety attachment and was named “Anxiety Attachment Level.” The second factor (6 items, loadings: 0.50–0.93) represented avoidance attachment and was named “Avoidance Attachment.” Together, these factors explained 53.7% of the variance.

The scale demonstrated good reliability, with Cronbach’s alpha ranging from 0.85 to 0.87. Responses were recorded on a 7-point Likert scale (1 = strongly disagree, 7 = strongly agree). Given that the primary aim of this study was to examine how pet companionship relates to well-being among middle-aged and older adults, we focused on participants’ attachment style toward their pets. Both anxious and avoidant attachment dimensions were measured; however, only anxious attachment was included as a moderator in the analysis. This decision was made to align with the study’s focus on emotional salience in human–animal interactions. Participants with avoidant attachment tendencies were not excluded from the sample but were not included in the moderation model.

### Data analysis

This study conducted data analysis using SPSS 23.0 statistical software, which included descriptive statistics and hierarchical regression analysis. Descriptive statistics were used to calculate correlations, means, and standard deviations among the questionnaire variables to understand the data distribution. The purpose of regression analysis is to examine whether there is a significant relationship between independent and dependent variables. Regression analysis serves both explanatory and predictive purposes. In this study, hierarchical regression was employed to analyze moderating effects and test hypotheses. Following Aiken et al.‘s [80] approach, group mean centering was used to address collinearity issues, which involves subtracting the mean from each independent and moderator variable and then multiplying them to obtain the product.

In the hierarchical regression, control variables were entered in the first step, including demographic variables (gender, age, marital status, retirement, etc.) and avoidance attachment. In the second step, independent variables (pet interaction) and moderator variables (neuroticism, anxiety attachment level) were added. In the third step, interaction terms between independent variables and moderator variables were included. Finally, the study examined whether there were interactions between independent and moderator variables. In the results figure of the moderating effects, the study used one standard deviation above the mean plus the mean as the “long interaction time” and one standard deviation below the mean minus the mean as the “short interaction time.” For the moderator variables, “high neuroticism” was one standard deviation above the mean, and “low neuroticism” was one standard deviation below the mean. Similarly, “high anxiety attachment” was one standard deviation above the mean, and “low anxiety attachment” was one standard deviation below the mean.

## Result

### Descriptive statistics

The detailed basic information of the participants is shown in Table 1. Then, Table 2 presents the descriptive statistics for the continuous variables in this study, showing the maximum, minimum, mean, and standard deviation to illustrate the data distribution. In the section regarding age and basic keeping pets information, the sample in this study had an age distribution ranging from 45 to 104 years old (mean age = 57.35, standard deviation = 7.24). At the time of the survey, the number of dogs currently kept by participants ranged from 1 to 9, while the number of cats reached up to 29. On average, middle-aged and older adults had more cats than dogs (average dogs = 1.57, average cats = 3.09). The age of pets ranged from newborn to 20 years old, and the duration of ownership varied widely, spanning from a few weeks (recently acquired) to several years (most of their lives).

**Table 1:** Demographic and pet-related information of participants

*N* = 232	Group	*N*	%
Gender	Male	38	16.4
	Female	194	83.6
Age	45–64 years old	199	85.8
	65 years old and above	33	14.2
Retirement status	Not retired yet	140	60.3
	Retired	92	39.7
Companion	Have	203	12.5
	None	203	87.5
Keeping dog(s)	Have	156	67.2
	None	76	32.8
Keeping cat(s)	Have	109	47.0
	None	123	53.0

**Table 2:** Descriptive statistics

Variables	Dimension	Min	Max	M	SD
	Age	45	104	57.35	7.24
Situation of keeping pets	Number of dogs raised	1	9	1.57	1.33
	Dog’s age (years)	0.3	18.0	9.00	4.50
	How long have you kept dogs (years)	0.3	52.0	9.72	8.18
	Number of cats raised	1	29	3.09	3.82
	Cat’s age (years)	0.3	20.0	7.42	5.23
	How long have you kept cats (years)	0.2	20.0	7.88	11.06
Pet interaction	Interactive Care	1.00	7.00	3.44	1.69
	Interactive Companionship	1.00	7.00	6.51	1.03
	Non-Interactive Companionship	1.00	7.00	5.57	1.98
	Pet interaction (Overall)	1.00	7.00	6.14	1.22
Attachment	Avoidance attachment	1.00	7.00	2.71	1.43
	Anxious attachment	1.00	7.00	1.60	0.95
Neuroticism		1.00	7.00	2.90	1.26
Well-being	Self-Growth and Acceptance	2.50	7.00	6.10	0.97
	Positive Relations with Others	1.33	7.00	5.63	1.18
	Purpose in Life	1.00	7.00	5.49	1.14
Interactive effect	Pet interaction * Neuroticism	−6.41	5.57	−0.16	1.30
	Interactive Care * Anxious attachment	−6.29	15.29	0.19	2.48

Note: Interaction effect is the cross-product of two variables after subtracting their means

On average, middle-aged and older adults spent less time on interactive caregiving (mean = 3.44), while the average scores for the other types of interactions (Interactive Companionship and Non-Interactive Companionship) were between 5 and 7. Concerning pet attachment, both avoidance attachment and anxiety attachment levels ranged from 1 to 7. On average, middle-aged and older adults had a slightly higher level of avoidance attachment to their pets (mean = 2.71) compared to anxiety attachment (mean = 1.60). Regarding well-being, the highest average was found in self-growth and acceptance (mean = 6.10), with the smallest variation in the sample (standard deviation = 0.97). The average for positive relationships with others (5.63) and life goals (5.49) was slightly lower than self-growth and acceptance, with standard deviations of 1.18 and 1.14, respectively, indicating some variation among the participants.

### Correlation analysis

Table 3 presents the correlation matrix. From Table 3, it is evident that there is no significant correlation between age and the three dimensions of well-being. Therefore, in subsequent analyses, age was not included as a control variable for well-being. In terms of keeping pets among middle-aged and older adults, there were more dog parents (*N* = 150) compared to cat parents (*N* = 116). Dog parents had a higher level of interactive caregiving (*r* =.57, *p* <.01), while cat parents had less interactive caregiving (*r* = −.48, *p* <.01), suggesting that cat parents were less likely to engage in interactive caregiving behaviors. However, there was no significant association between keeping cats or dogs and the three dimensions of well-being.

**Table 3:** Correlation analysis

Variables	Dimension	7	8	9	10	11	12	13	14	15	16
Demographic variables	1. Age	0.04	− 0.22^**^	− 0.20^**^	− 0.24^**^	− 0.02	0.38^**^	− 0.08	− 0.03	0.13	0.03
	2. Gender (male = 1, female = 0)	0.04	− 0.11	− 0.22^**^	− 0.20^**^	− 0.04	− 0.09	− 0.02	− 0.15^*^	− 0.03	0.05
	3. Companion (have = 1, none = 0)	0.02	− 0.10	− 0.12	− 0.13	0.11	0.06	0.14	0.01	0.14^*^	0.13^*^
Keeping dog(s) or cat(s)	4. keeping dog(s) (have = 1, none = 0)	0.57^**^	0.03	0.12	0.09	0.07	− 0.05	− 0.10	− 0.08	0.02	0.01
	5. keeping cat(s) (have = 1, none = 0)	− 0.48^**^	− 0.03	− 0.12	− 0.09	− 0.06	0.04	0.10	0.04	− 0.03	0.06
	6. Number of pets raised	− 0.08	0.08	− 0.02	0.03	− 0.08	− 0.12	− 0.07	0.06	− 0.02	0.03
Pet interaction	7. Interactive Care	-									
	8. Interactive Companionship	0.28^**^	-								
	9. Non-Interactive Companionship	0.20^**^	0.48^**^	-							
	10. Pet interaction (Overall)	0.27^**^	0.82^**^	0.90^**^	-						
Attachment	11. Anxious attachment	0.08	0.04	0.10	0.09	-					
	12. Avoidance attachment	− 0.15^*^	− 0.63^**^	− 0.44^**^	− 0.61^**^	0.18^**^	-				
Neuroticism	13. Neuroticism	− 0.06	− 0.09	− 0.10	− 0.10	0.35^**^	0.21^**^	-			
Well-being	14. Self-Growth and Acceptance	0.08	0.19^**^	0.27^**^	0.27^**^	0.00	− 0.24^**^	− 0.24^**^	-		
	15. Positive Relations with Others	0.13^*^	0.04	0.10	0.08	0.08	0.01	− 0.18^**^	0.54^**^	-	
	16. Purpose in Life	0.05	0.04	0.04	0.05	− 0.10	− 0.13	− 0.34^**^	0.59^**^	0.60^**^	-
Interactive effect	17. Pet interaction (Overall) * Neuroticism	0.08	0.17^*^	0.14^*^	0.17^**^	0.07	− 0.26^**^	0.20^**^	− 0.13^*^	− 0.03	− 0.13^*^
	18. Interactive Care * Anxious attachment	− 0.03	0.03	0.00	0.02	0.19^**^	0.13	0.12	− 0.01	− 0.14^*^	− 0.06

Note: Interaction effect is the cross-product of two variables after subtracting their means, * p < .05. ** *p* < .01

Furthermore, since “interactive companionship” and “non-interactive companionship” had similar associations with the three dimensions of well-being, both showing significant positive correlations with “self-growth and acceptance” (interactive: *r* =.19, *p* <.01, non-interactive: *r* =.27, *p* <.01), no significant correlation with “positive relationships with others” (interactive: *r* =.04, non-interactive: *r* =.10), and no significant correlation with “life goals” (interactive: *r* =.04, non-interactive: *r* =.04), these two types of companionship were combined into one category named “companionship” for the sake of simplicity in subsequent analysis.

Regarding pet attachment, avoidance attachment was significantly negatively correlated with “self-growth and acceptance” in well-being (*r* = −.24, *p* <.01). Therefore, in the subsequent analysis of moderation effects, avoidance attachment was used as a control variable in the model. However, anxiety attachment level did not show a significant association with the dimensions of well-being. Consequently, regression analysis was conducted to explore whether it moderates the relationship between pet interaction time and well-being.

### Pet-related and demographic variables in predicting psychological well-being

To further examine the potential role of pet-related and demographic variables in predicting psychological well-being, we conducted a set of multiple regression analyses using gender, age, companion (have/none), and number of pets as predictors of the three dimensions of well-being.

As shown in Table 4, gender significantly predicted Self-Growth and Acceptance (*β* = –0.15, *p* <.05), with females reporting higher scores than males. Companion was positively associated with Positive Relations with Others (*β* = 0.15, *p* <.05), and Neuroticism (*β* = 0.14, *p* <.05). However, the number of pets raised did not significantly predict any of the three well-being outcomes.

**Table 4:** The effects of demographic variables on main variable(s)

	Self-Growth and Acceptance		Positive Relations with Others		Purpose in Life		Neuroticism	
	*β*	*VIF*	*β*	*VIF*	*β*	*VIF*	*β*	*VIF*
Gender (male = 1, female = 0)	− 0.15^*^	1.03	− 0.06	1.03	0.03	1.03	− 0.04	1.03
Age	− 0.02	1.01	0.14	1.01	0.03	1.01	− 0.08	1.01
Companion (have = 1, none = 0)	0.03	1.03	0.15^*^	1.03	0.13	1.03	0.14^*^	1.03
Number of pets raised	0.06	1.01	− 0.002	1.01	0.04	1.01	− 0.06	1.01
*ΔR* ^*2*^	0.08		0.02		0.003		0.013	
Sig. *F* Change	0.205		0.049		0.330		0.133	

^*^p <.05

### Results of the moderation effect of neuroticism

Table 5 presents the results of neuroticism moderating the relationship between overall pet interaction time and self-growth and acceptance. A hierarchical regression analysis was conducted to examine whether neuroticism moderated the relationship between pet interaction and self-growth and acceptance. From Table 5, it can be observed that pet interaction (*β* = 0.20, *p* <.01) and neuroticism (*β* = −0.18, *p* <.01) both have main effects on self-growth and acceptance. A significant moderating effect was observed between pet interaction and neuroticism on self-growth and acceptance (*β* = −0.13, *p* <.05).

**Table 5:** Moderating effects of neuroticism on the relationship between pet interaction and self-growth and acceptance

ΔR^2^	Dependent Variable: Self-Growth and Acceptance			
	Model A (β)	Model B (β)	Model C (β)	VIF
Step 1 (*ΔR*^*2*^ = 0.08; Sig. *F* Change < 0.000)				
Gender	−0.14^*^	− 0.11	−0.12^*^	1.01
Avoidance attachment	−0.24^***^	−0.9	−0.12	1.01
Step 2 (*ΔR*^*2*^ = 0.13; Sig. *F* Change < 0.000)				
Pet interaction (Overall)		0.20^**^	0.21^**^	1.60
Neuroticism		−0.18^**^	−0.15^*^	1.06
Step 3 (*ΔR*^*2*^ = 0.14; Sig. *F* Change = 0.102)				
Pet interaction (Overall) * Neuroticism			−0.13 ^*^	1.16

Note: The values in the table represent unstandardized regression coefficients (*β*)

^*^p< .05.^**^ p < .01.^***^p< .001

**Fig. 1:**
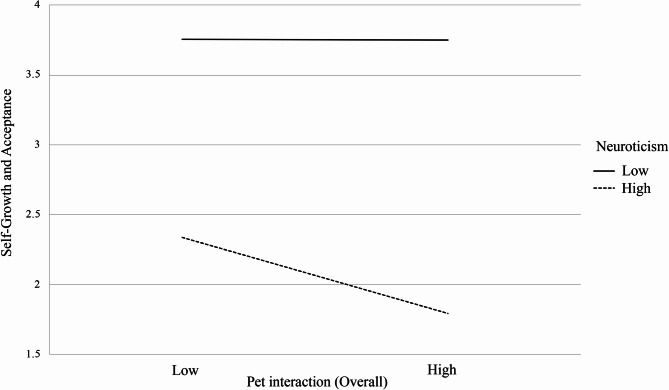
Moderating effects of neuroticism on the relationship between pet interaction and self-growth and acceptance

Figure 1 illustrates the results of neuroticism moderating pet interaction and self-growth and acceptance. From Fig. 1, it can be seen that middle-aged and older individuals with low neuroticism have higher self-growth and acceptance compared to those with high neuroticism. Middle-aged and older individuals with low neuroticism maintain consistent levels of self-growth and acceptance regardless of the length of time spent with their pets (3.76 ~ 3.76). However, for middle-aged and older individuals with high neuroticism, their self-growth and acceptance decreases when the pet interaction time is longer (2.34 ~ 1.79).

### Results of the moderation effect of anxiety attachment

Table 6 presents the results of anxiety attachment moderating the relationship between interactive care and positive relationships with others. A hierarchical regression analysis was conducted to examine whether anxiety attachment moderated the relationship between interactive care and positive relations with others. From Table 6, it can be seen that interactive care (*β* = 0.15, *p* <.05) has significant main effects on self-growth and acceptance. There was a marginally significant interaction between interactive care and anxious attachment (*β* = −0.11, *p* =.056), indicating a potential trend that did not reach conventional levels of statistical significance.

**Table 6:** Moderating effects of anxious attachment on the relationship between interactive care and positive relations with others

ΔR^2^	Dependent Variable: Positive Relations with Others			
	Model A (β)	Model B (β)	Model C (β)	VIF
Step 1 (*ΔR*^*2*^ = 0.01; Sig. *F* Change = 0.415)				
Companion (have = 1, none = 0)	0.10	0.09	0.09	1.00
Step 2 (*ΔR*^*2*^ = 0.23; Sig. *F* Change < 0.000)				
Interactive care		0.15^*^	0.14^*^	1.01
Anxious attachment		0.08	0.10	1.02
Step 3 (*ΔR*^*2*^ = 0.25; Sig. *F* Change = 0.624)				
Interactive care * Anxious attachment			−0.11^†^	1.04

Note: The values in the table represent unstandardized regression coefficients (β)

^†^*p* < .10. ^*^p < .05

**Fig. 2:**
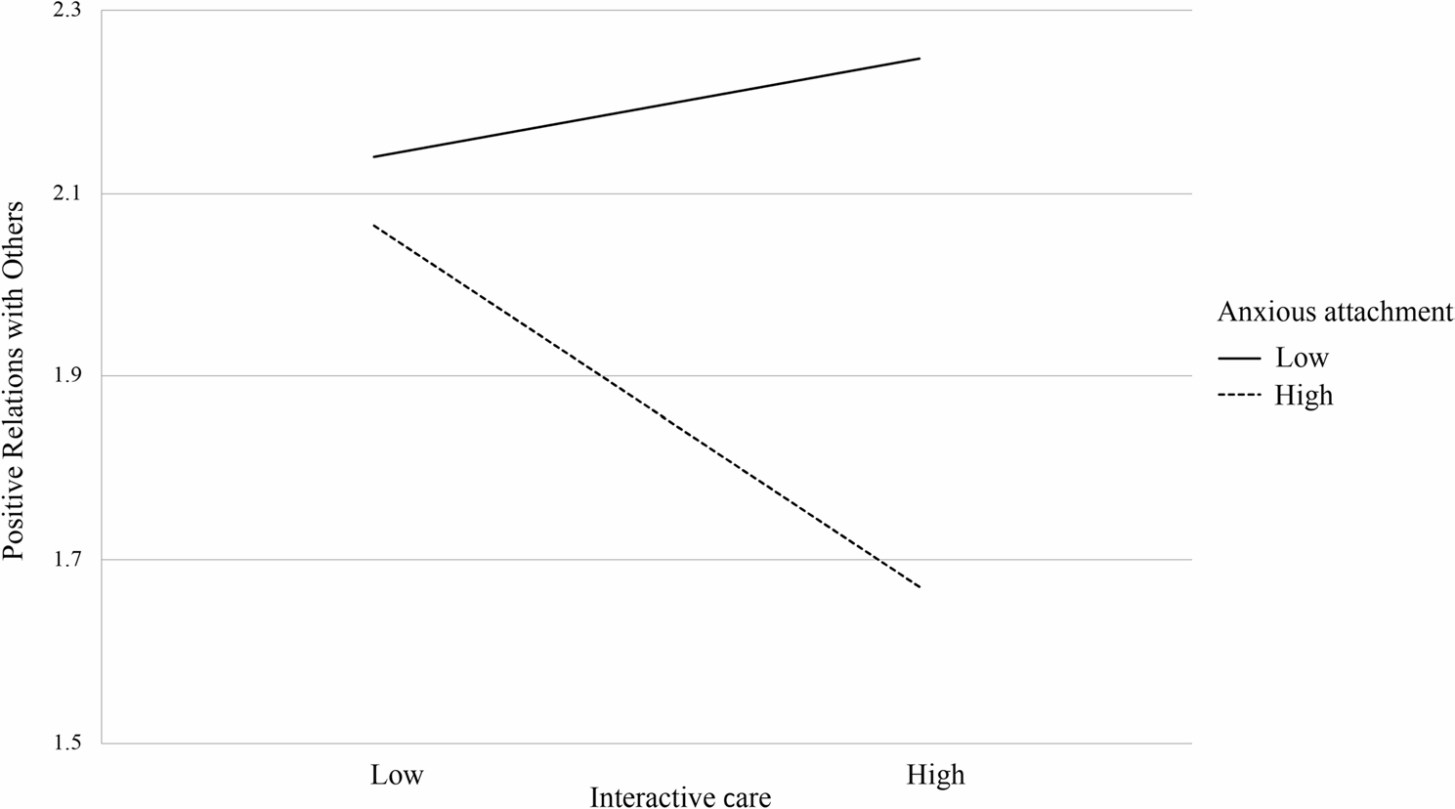
Moderating effects of anxious attachment on the relationship between interactive care and positive relations with others

Figure 2 illustrates the trend of anxious attachment moderating the relationship between interactive care and positive relationships with others. From Fig. 2, it can be observed that middle-aged and older individuals with low anxiety attachment have higher positive relationships with others compared to those with high anxiety attachment. When middle-aged and older individuals spend more time interacting with pets, those with low anxiety attachment experience an increase in their positive relationships with others (2.14 ~ 2.25). In contrast, those with high anxiety attachment experience a decrease in positive relationships with others (2.06 ~ 1.67) as they spend more time caring for their pets. In other words, when middle-aged and older individuals spend only a short amount of time caring for their pets, the difference in positive relations with others between those with high (*M* = 2.06) and low (*M* = 2.14) anxiety attachment is minimal, with a mean difference of just 0.08. However, with increased time spent on pet caregiving, the difference in positive relations with others between high (M = 1.67) and low (M = 2.25) anxiety attachment groups becomes more pronounced, showing a notable gap of 0.58.

## Discussion

This study aimed to examine the relationship between pet companionship and psychological well-being among middle-aged and older adults, with particular attention to the moderating roles of personality traits and attachment styles. Using a cross-sectional survey design, we assessed how companionship time, neuroticism, and anxiety-based attachment to pets jointly influence three dimensions of well-being. The results indicated that while pet companionship time alone was not a strong predictor of well-being, its effects varied depending on individual differences—particularly neuroticism and anxious attachment tendencies. These findings offer a more nuanced understanding of how human–pet relationships relate to psychosocial outcomes among middle-aged and older adults.

### Moderating effect of neuroticism

The results of this study show that, among individuals who spend more time caring for their pets, those with higher neuroticism scores report significantly lower levels of well-being compared to those with lower neuroticism scores. In particular, as time spent on pet caregiving increases, individuals with high neuroticism demonstrate a noticeable decline in Self-Growth and Acceptance, whereas individuals with low neuroticism show relatively stable well-being levels. Pet companionship includes a range of daily interactions such as talking to pets [105], petting them, or having them accompany their owners during work [106] or rest. While these behaviors are often viewed as emotionally supportive, personality traits like neuroticism may influence how such interactions are perceived and processed. For example, Curb et al. [58] suggest that pet guardians with higher neuroticism may exhibit more controlling or reactive behaviors, which are associated with lower pet satisfaction. This may be particularly relevant in households with dogs or cats that display behavioral challenges [48]. Over time, these patterns could contribute to increased emotional strain in pet caregiving, especially for those with heightened emotional sensitivity. Although the current study does not directly examine the mechanisms underlying these outcomes, the findings point to the importance of considering individual personality traits in understanding how pet companionship affects well-being.

While pet companionship might be emotionally meaningful, extended time in caregiving roles may place additional emotional demands on individuals, particularly those who are more prone to negative affectivity. Given that neuroticism is negatively associated with well-being, individuals with higher neuroticism tend to experience greater difficulties with emotional regulation [81, 82] and maintaining social relationships [83], which may contribute to lower overall well-being. Accordingly, the moderation analysis in this study showed that well-being decreased more sharply among individuals with high neuroticism as their pet companionship time increased, whereas individuals with low neuroticism exhibited a more stable pattern.

Our results showed that neuroticism moderated the relationship between time spent with pets and self-growth and acceptance, but did not significantly moderate the relationship between time spent with pets and positive relations with others. Neuroticism can negatively predict positive relationships with others [83]. Still, interactions between pet parents and their pets differ from human interactions, as pet parents’ attitudes during interactions with pets involve caregiving and play. Through the process of caring for and spending time with their pets, pet parents increase life satisfaction and alleviate stress [84]. Human-to-human interactions encompass a broader and more complex range of factors [112]. Therefore, while individuals with high neuroticism may have negative relationships with others, this does not extend to their interactions with pets, and neuroticism does not moderate the effect in this context.

### Moderation effect of anxiety attachment

The results of this study indicate that attachment anxiety fully moderates the association between middle-aged and older adults’ interactive caregiving of pets and their positive relationships with others. When caregiving time is short, well-being remains unaffected by attachment anxiety. However, when caregiving time is prolonged, a significant difference emerges between individuals with high and low attachment anxiety. Those with low attachment anxiety experience an increase in well-being, while those with high attachment anxiety see a decrease. This is consistent with past research showing that attachment between people and pets can impact the lives of both parties [95–97]. While much of the literature on attachment anxiety stems from studies on human–human relationships, these findings offer insights that may be relevant in human–animal contexts. For example, Overall et al. [85] found that individuals with attachment anxiety often perceive threats to close relationships in an exaggerated way, which may induce feelings of guilt in their partners. Although this finding comes from interpersonal contexts, it is possible that middle-aged and older adults with attachment anxiety exhibit similar emotional patterns when caring for pets, leading to increased stress or reduced well-being over time [86]. Furthermore, middle-aged and older adults with attachment anxiety tend to be particularly sensitive to threats and are more inclined to hold negative views of external groups [87]. This may make it difficult for them to develop trustful emotions towards their pets in their interactions.

The relationship between attachment anxiety and well-being has been documented in both human interpersonal contexts and human–animal interactions, which aligns with the findings of this study. For example, Green [88] observed that pet parents with high levels of attachment anxiety tend to closely monitor their pets’ behaviors and seek frequent feedback or responses. While these behaviors may reflect strong emotional investment, they can also contribute to increased tension or stress during interactions, particularly when expectations are not met. Moreover, anxious attachment pet parents frequently doubt their pets’ affection, necessitating reassurance and fearing a loss of trust if they leave their pets alone at home [59]. On the other hand, pet parents with anxious attachment may report higher levels of caregiving and attentiveness, which could have unintended negative consequences for their pets. For instance, individuals with high attachment anxiety might offer excessive treats or toys as a form of reassurance or compensation, potentially leading to overstimulation or weight gain in their pets [107]. Maladaptation in interpersonal relationships also reduces the well-being derived from keeping pets [57].

## Limitations and suggestions

### Limitations of data analysis

First, although age was considered a relevant factor in prior studies, our analysis indicated that it was not significantly correlated with the outcome variables, and thus it was not included as a covariate. However, future studies with larger and more diverse samples could explore the role of age more comprehensively, particularly in relation to its potential moderating effects [89].

Second, our study combined interactive and non-interactive companionship into a single construct based on theoretical and empirical considerations. While this approach provided a parsimonious analytical model, we acknowledge that treating these constructs separately might yield additional insights [90, 91]. Future research could examine the distinct contributions of each companionship type to deepen the understanding of their respective psychological mechanisms.

Third, our moderation models were designed based on theoretical justifications, rather than an exhaustive exploratory approach. While we recognize that moderation effects can sometimes emerge even in the absence of significant zero-order correlations, testing all possible moderation models without a strong theoretical foundation increases the risk of Type I errors [92]. Future studies could expand on our findings by systematically testing additional moderation models, particularly in studies with larger sample sizes and more robust theoretical frameworks [93].

Fourth, while our findings suggest that different pet ownership experiences may be associated with distinct behavioral patterns, our sample size limited our ability to conduct separate subgroup analyses for different pet owner types. Future research could adopt larger and more balanced samples to explore whether pet-specific interactions exhibit unique behavioral patterns [94].

### Difficulty in participant recruitment

This study encountered several challenges in recruiting participants. Eligibility criteria required participants to be adults aged 45 and above and to currently keep a dog or a cat. These two criteria may have contributed to recruitment difficulties, as there are relatively few recruitment channels that simultaneously reach both middle-aged and older adults and current pet guardians. For example, research in Taiwan targeting older adults has traditionally recruited from senior learning centers [e.g., 108, 109], or, in the case of studies like Liu et al. [110] ’s on older blood donors, directly from institutions offering related services. In contrast, this study lacked a clearly defined setting or platform where a concentrated population of middle-aged and older pet guardians could be accessed efficiently. During the pilot phase, researchers attempted on-site recruitment in public spaces, such as parks, sidewalks, and veterinary clinics, to identify potential participants. However, determining whether middle-aged and older adults were pet guardians proved difficult in these contexts—especially when individuals were not accompanied by their pets. Even when participants were encountered at veterinary clinics, they were often in a state of anxiety or distress while waiting for their pets to receive care, making it ethically inappropriate to interrupt them. While strategies such as distributing pamphlets or flyers in these locations were considered, researchers found that such passive recruitment approaches yielded low engagement during initial trials and were not pursued further due to limited project resources. Out of empathy, researchers were reluctant to disturb them while they waited for their pets to receive medical attention. At pet grooming salons, pet guardians were usually in a hurry and did not remain on-site during the grooming process. These factors made it challenging to distribute paper-based surveys and contributed to difficulties in reaching the required number of participants. To address these limitations, electronic surveys were distributed through Facebook, including both Facebook groups and Facebook Messenger shared among friends and family. This approach made it easier to reach eligible participants and allowed them to complete the survey at their convenience. As a result, both the pilot and formal surveys in this study were administered using electronic Google Forms.

### Difficulty in measuring pet interaction

Regarding the measurement of length of pet interaction in this study, researchers encountered several difficulties during the pilot phase when using open-ended questions to ask about daily caregiving time (in minutes) and frequency, as well as daily companionship time (in minutes) and frequency. Most participants found it challenging to provide accurate estimates, as they spent much of the day with their pets and were unable to calculate precise durations or frequencies. Many simply responded with vague terms such as “many times.” Furthermore, participants had differing personal definitions of “caregiving” and “companionship,” which made it difficult to standardize responses across the sample. To address these issues, and due to time and resource constraints, the formal survey adopted a structured pet interaction scale. Researchers first consulted friends and acquaintances who kept cats or dogs to identify common pet-related behaviors. These behaviors were then categorized and labeled using exploratory factor analysis. While this approach offered a more feasible and structured way to measure pet interaction, it may still introduce potential biases. For example, the behaviors included may reflect a limited range of interaction types that are more common in certain social or cultural contexts. Additionally, self-reporting remains subject to individual interpretation and recall bias. Future research could develop more comprehensive and validated tools to capture pet interaction patterns in greater depth.

In addition, one of the limitations of this study concerns the reliability of the “interactive care” subscale, which had a Cronbach’s alpha of 0.59, just below the commonly accepted threshold for internal consistency. While we have included this data in our analyses, it is important to note that the lower reliability of this subscale may influence the interpretation of the results. Future research should consider refining the “interactive care” subscale by adding more items or improving item clarity to enhance its reliability. Additionally, researchers should remain cautious when interpreting the results from this subscale, as they may deviate from the findings of other subscales with higher reliability.

Last but not least, the current literature provides limited evidence on how the number of pets affects well-being. This makes it difficult to construct assumptions or hypotheses without a stronger research foundation. Future studies could explore this variable further, as it represents a potential avenue for deepening our understanding of the complex dynamics between pet guardianship and well-being. This study recommend that future research investigate the number of pets raised as an important factor that may influence the well-being of pet owners, particularly middle-aged and older adults.

### Suggestions

The well-being scale used in this study was reduced to three dimensions through factor analysis: self-growth and acceptance, positive relationships with others, and life goals. It’s suggested that future studies consider adding variables such as social support and loneliness to the dependent variables. Additionally, exploring the significant differences in caregiving between dogs and cats could be a focus of future research. Furthermore, this study only used extraversion and neuroticism traits from the Big Five personality traits and attachment anxiety as moderator variables. This doesn’t comprehensively explain the connection between pet interaction and well-being. Future research could delve deeper into the interaction between middle-aged and older adults and one specific type of pet or use other variables for mediation or moderation analyses to gain a more comprehensive understanding.

## Conclusion

More and more middle-aged and older adults view pets as important companions. This trend has been exacerbated by the COVID-19 pandemic, which has increased the time middle-aged and older adults spend at home. Having a pet can reduce loneliness, increase physical activity, and provide a sense of accomplishment. This study shows that keeping a pet doesn’t automatically lead to improved physical and mental health for middle-aged and older adults. Understanding the personality traits of middle-aged and older adults and their suitability for a particular type of pet, as well as how they interact with their pets, is essential. Middle-aged and older adults should also allocate time to their own lives and social interactions, as pets, while providing companionship, cannot replace human interaction. By balancing life goals, social support, and learning, middle-aged and older adults can find that keeping pets can promote physical and mental health and increase well-being.

## Data Availability

The datasets generated and/or analyzed during the current study are not publicly available due to restrictions their containing information that could compromise the privacy of research participants, but are available from the corresponding author on reasonable request.

## Abbreviations

M: Mean
SD: Standard deviation
EFA: Exploratory factor analysis
VIF: Variance inflation factor
KMO: Kaiser-meyer-olkin
